# Effect of Geolocators on Migration and Subsequent Breeding Performance of a Long-Distance Passerine Migrant

**DOI:** 10.1371/journal.pone.0082316

**Published:** 2013-12-04

**Authors:** Debora Arlt, Matthew Low, Tomas Pärt

**Affiliations:** Department of Ecology, Swedish University of Agricultural Sciences, Uppsala, Sweden; Institute of Ecology, Germany

## Abstract

Geolocators are small light-weight data loggers used to track individual migratory routes, and their use has increased exponentially in birds. However, the effects of geolocators on individual performance are still poorly known. We studied geolocator effects on a long-distance migrating passerine bird, the northern wheatear (*Oenanthe oenanthe* L.). We asked the general question of whether geolocators affect migratory behaviour and subsequent reproductive performance of small passerines by comparing arrival time, breeding time, breeding success and survival of geolocator versus control birds of known identity and breeding history. During two years geolocator birds (n=37) displayed a lower apparent survival (30%) as compared to controls (45%, n=164). Furthermore, returning geolocator birds (n=12) arrived on average 3.5 days later, started laying eggs 6.3 days later, and had lower nest success (25%) than control birds (78%). Our results suggest that geolocators affect migratory performance with carry-over effects to the timing of breeding and reproductive success in the subsequent breeding season. We discuss the implications of such geolocator effects for the study of migratory strategies of small passerines in general and suggest how to identify and investigate such effects in the future.

## Introduction

Archival light geolocators are data-logging devices that allow inferring geographical locations from time-specific data on light intensity levels [1-4]. Compared to satellite tracking, geolocation by light is less accurate, but the devices are smaller, lighter and cheaper. Geolocators have become increasingly popular for tracking migratory routes of birds, especially passerines (e.g. [5,6]), that are too small for other larger tracking devices. The ability to track individual migration routes opens unprecedented opportunities to reveal not only wintering areas, migratory routes and connectivity (e.g. [7-9]), but also migratory time schedules, including individual departure and migration decisions (e.g. [10-12]). Detailed knowledge on the migratory strategies of a range of species is important for understanding the ecology and evolution of migration [13,14], conservation of migratory species [15,16] and effects of climate change on these populations.

Tracking devices can, however, have negative consequences [17], although how geolocators may influence individual behaviour and survival are poorly known. Geolocator studies usually assume that increases in flight costs are small and refer to the suggested upper permissible load limits for radio-transmitters of 5% of the bird’s body mass [18]. A recent meta-analysis of the effects of all types of tracking devices (i.e. data loggers, radio and satellite transmitters; not including geolocators but devices of comparable design) on birds found generally small but significant negative effects on a variety of behavioural and ecological parameters; the most substantial being increased energy expenditure [17]. Recent aerodynamic studies of backpack-style mounted transmitters or loggers suggest that increased drag may be at least as important as the additional weight of the device in increasing flights costs [19,20]. Such energetic costs could result in significant increases in daily energy expenditure [19], potentially influencing survival and migration performance, or induce carry-over effects [21] on breeding performance. Thus, if geolocators are going to be widely used to study migration, potential biases from their use need to be well understood. 

Because migratory behaviour and physiology are difficult to examine without tracking devices, there are no direct data on the effects of these devices on migratory performance in birds. Nevertheless, we can make inferences from parameters that are thought to be affected by migratory performance, e.g. arrival and breeding time. Arrival time is likely related to departure time, migration speed and distance from the wintering quarters and the timing of breeding is likely influenced by the effort spent during migration and condition at arrival [22]. Here, we compare return rates, post-migratory arrival and breeding time and reproductive performance in northern wheatears (*Oenanthe oenanthe*; hereafter ‘wheatears’) fitted with geolocators (n=39; of which 12 returned after migration), to individuals from our study population only marked with traditional leg rings. Thus, we could compare the fate of individuals with a known breeding history. Wheatears are long-distance migrants, breeding across most of the Palearctic and wintering in sub-Saharan Africa [23]. From their breeding grounds in southern central Sweden, wheatears begin their migration in August and return in the following April-May. Because increased energy expenditure during nestling food provisioning may reduce survival to the next year (e.g. [24]), it is plausible that increased energy expenditure associated with carrying geolocators would also influence return rates and migration behaviour of passerines such as wheatears. Thus, we investigated whether there were differences between wheatears fitted with geolocators and control birds in the post-deployment year regarding (1) return rates, (2) timing of arrival to the breeding grounds, (3) timing of breeding, and (4) reproductive performance.

## Materials and Methods

### Ethics statement

This study was carried out in accordance to the legal and ethical requirements for animal research (approved by the ethical committee (Uppsala Djurförsöksetiska nämnd) at the district court of Uppsala, permit no. C117/8). Geolocators had a relative load of approximately 5% of the birds' body mass (Table 1), i.e. the suggested upper permissible load limit [18]. The permit for ringing and use of traps and mist nest was issued by the Swedish Bird Ringing Centre (permit no. 509). Birds were trapped in approved traps or mist nets, handled and measured according to approved methods, and handling time was minimised to ameliorate potential suffering. We had permission by the land owners for access to privately owned land.

**Table 1:** Geolocator types (British Antarctic Survey, Cambridge, UK) used during 2010 and 2011.

	2010	2011
model	Mk12S	Mk20ASLT
thickness (mm)	5.5	5.5
stalk length (stalk angle)	15(30)	10(25)
weight (g; including harness)	1.1(1.3)	0.8(1.0)
mean body mass (g) ±SD	22.9±1.0	23.5±1.4
mean load (% of body mass)	5.7	4.3
maximum load (% of body mass)	6.1	4.9

Mean and maximum relative load, i.e. total tag weight in % of mean and minimum, respectively, body weight of the tagged individuals at the time of deployment (during chick feeding when body mass is low; [30]).

### Study population

We studied a population of wheatears breeding in southern central Sweden (59°50’N, 17°50’E) in an area of about 60 km^2^. Wheatears arrive in mid-April to mid–May, the first pairs start egg laying in early May, and the majority of nestlings fledge before mid-June (details in [25-27]). After post-breeding moult the birds start their migration to Africa in August. Each year we monitored all sites potentially suitable for wheatears from early April to the end of June. Detailed breeding data were collected in a 40 km^2^ central part of the study area. Nearly all (97%) males and 76% of all females could be aged as either young (one year old) or older (≥ two years old) based on plumage characteristics (see [25]). Each year we uniquely colour-ringed many adults, resulting in 70-75% of breeding males and females being marked at the end of the breeding season. Birds were weighed to the nearest 0.1 g, and tarsus length is measured to the nearest 0.1 mm. Apparent survival was estimated for birds breeding in the 40 km^2^ core area by return to the entire 60 km^2^ area in subsequent years. In the outer part of the study area we monitored site occupancy, nest success and identity of the breeding birds; this allowed us to limit biases in detection from between-year dispersal because we monitored all individuals dispersing within 2 km from the core area (adults disperse short distances between breeding seasons: median distance males: 308 m, females: 352 m [27]). Thus, annual re-sighting probability was high (males: 0.98, females: 0.89 [24]).

Nest success was recorded as successful or failed. A breeding attempt was defined successful when we observed fledglings or heard intense warning calls of the parents after fledging [25]. Nest failures, on average 30%, were mostly due to predation [25]. Data on nest success were missing when the nest had not been visited at or after the time of fledging (about 12% of all breeding attempts). The number of fledged offspring was defined as the number of chicks ringed minus number of dead chicks found in the nest after fledging, and was missing when the nest could not be accessed.

Monitoring was normally done every third to fifth day, but every second day during the arrival period. Arrival date was defined as the date when we observed an individual for the first time in the study area. Arrival dates were thus estimated with an accuracy of 2-4 days. Breeding time was defined as the date the first egg was laid. Egg laying dates were either calculated based on the age of chicks in the accessible nest (80-85% of all dates, accuracy of 0-1 days), or by observations of breeding behaviour that could be used to establish likely time intervals for egg laying dates (e.g. nest building, first observation of feeding parents, age of fledged young; 15-20%, most interval lengths ≤5 days). 

In our study area wheatears breed in a mosaic of farmland habitats (pastures, farmyards, crop fields, unmanaged grassland [28]). Territories were characterised by vegetation structure describing the height of the ground vegetation layer (field layer height). Territories were categorised as having either a short (<5 cm throughout the breeding season) or tall field layer (growing >15 cm during late incubation and nestling care [25,26]). Territory field layer height is an important component of habitat quality with lower reproductive performance and adult survival in tall field layers [24-26].

### Geolocators

We deployed British Antarctic Survey geolocators on 39 wheatears (Table 2), using types Mk12S in 2010 and MK20ASLT in 2011 (Table 1). Birds for geolocation were chosen among pairs feeding young during early and mid-June, covering the variation of breeding times from early to late breeders. We chose birds of both sexes, excluding birds known to be >4 years old, and avoiding birds breeding in tall field layer territories because of a lower expected survival rate [24]. In eight cases both pair members were fitted with a geolocator. Wheatears were trapped when they were feeding young in the nest. A majority of the returning individuals with geolocators were re-trapped before nest building to retrieve the geolocator. Geolocators were attached using a Rappole-Tipton style harnesses [29] made from 1 mm elastic EPDM rubber cord (Polymax, http://www.polymax.co.uk/. Accessed 2013 May 10) and leg-loop length was adjusted individually to each bird. Ringing and geolocator fitting lasted 10-15 min, with an additional few minutes for taking basic measurements. This handling time is similar to our normal ringing and measuring procedure, after which individuals normally resume feeding their offspring within 30 min. All tagged individuals were observed at the same site after deployment. The geolocator was normally covered by mantle feathers and difficult to see in the field (Figure S1). Often only the tip of the stalk could be seen (Figure S1); on some individuals there was a slight bulge visible where feathers covered the geolocator. Total tag weight (geolocator and harness) resulted in a relative load of about 4-6% of the body weight of the tagged individuals (Table 1). While little fat is stored during the breeding season, on migration, wheatears regularly increase their body mass by about 40-100% of their fat-free body mass [30].

**Table 2:** Number of geolocators deployed on male and female wheatears during 2010 and 2011, and number of tagged individuals that returned and were recaptured during the subsequent year.

		tagged individuals	returned individuals	recaptured individuals
2010	males	6	2	2
	females	4	3	2
	total	10	5	4
2011	males	13	2	2
	females	16	5	5
	total	29	7	7

We selected control birds based on the same criteria as used for selecting geolocator birds. We used only individuals that were trapped during the same years as geolocator birds, and in the same central parts of the study area as geolocator birds. As geolocator birds were tagged when feeding nestlings, we used only control birds which were feeding nestlings (i.e. excluding individuals with nest failures before hatching), and we excluded individuals >4 years old. Thus we chose control birds to match geolocator birds in terms of location, treatment (trapping and handling), parental effort and age to avoid biases in condition and in estimates of subsequent apparent survival rates and reproductive performance. Comparing geolocator and control birds in the deployment year still showed geolocator birds to be slightly heavier and to breed slightly earlier (Table 3). These differences, however, only make tests of negative effects of geolocators in the next year conservative (as one may expect early and heavy birds to be of high quality; and with respect to results for egg laying dates, see below). Arrival time, nest success and number of fledglings in successful nests did not differ between these two groups of birds in the deployment year (Table 3). Geolocator birds showed no obvious differences in flight or breeding behaviour from other birds in the weeks post-deployment.

**Table 3:** Comparison of individual and breeding parameters between geolocator (geo) and control (ctr) birds in the deployment year.

	control		geolocator		GLM
	mean±SE	n	mean±SE	n	median (CI)
body mass (g)	22.44±0.11	105	23.45±0.15	37	1.06 (0.61/1.51)
arrival date	0.85±0.72	78	0.34±0.71	34	-0.13 (-2.57/2.32)
egg laying date	1.09±0.65	87	-2.21±0.73	33	-3.00 (-5.43/-0.58)
nest success (%)	91.9	87	87.5	24	-0.67 (-2.32/1.14)
no. fledglings	5.0±0.2	57	5.1±0.2	16	

Differences of geolocator vs. control birds predicted by Bayesian GLMs are presented as median and 95% credible interval (CI; lower/upper bound) of the posterior distribution for the grouping variable (for analysis details see Methods). Presented mean±SE refer to raw data. Arrival and egg laying date were standardised for annual variation. Number of fledglings was considered only for successful nests.

In 2011 and 2012 we searched for returning individuals within our study area and at sites potentially suitable for wheatears in the surroundings within ca. 2 km from the borders of our study area. A total of 12 individuals returned (Table 2), all bred at territories with a median distance of 76 m from their previous year’s territory (min=0 m, max=1512 m). 

### Analyses

We tested whether there were differences between geolocator and control birds in terms of (1) apparent survival to the subsequent breeding season, and (2) arrival time at the breeding ground, (3) breeding time, and (4) reproductive performance following migration. Reproductive performance was investigated by analysing nest success. Because of low success rate data on number of fledglings for geolocator birds was available from only two nests, precluding us from analysing fledging success. We used generalised linear models (GLM) with Gaussian distribution, or binomial distribution with a logit link (for nest success and survival). 

Arrival and egg laying dates were standardised for annual variation (deviation from population mean in a given year). Within-individual changes in timing between consecutive years were calculated as the difference in dates between years, with positive values corresponding to a relative later date in the second year. When investigating arrival and breeding time, as well as within-individual changes between years, we accounted for the baseline value of the arrival or egg laying date in the deployment year by including a covariate expressing the deviation of an individual’s date from the population mean date in the deployment year [31]. Males generally arrive a few days before females, and old individuals arrive before young individuals [26,32], therefore we included sex and age class as covariates in analyses of arrival date. Several geolocator birds were recaptured prior to egg laying and the capture event could potentially cause delay the initiation of breeding. However, the difference in egg laying dates between geolocator and control birds (see Results) did not change when we restricted the geolocator birds to only those recaptured after clutch initiation, and we therefore present data based on all geolocator birds.

 Differences between geolocator and control birds in survival and reproductive performance were analysed including year as covariate. Survival analysis included sex, nest success and territory field layer (short vs. tall) since survival of wheatears in our study population differs between sexes, individuals with successful and failed breeding and individuals occupying territories of different field layer height [24]. Analyses of nest success include age class, territory field layer height and breeding time [25,26], and use breeding attempts as the unit of observation. Age class was the age class of the geolocator bird, or when both pair members had been tagged and for controls we randomly chose to use either the male’s or female’s age.

Sample sizes vary between analyses depending on missing data, especially for the larger control group (see Tables 3 and 4). All analyses were done in a Bayesian framework using a Gibb’s MCMC sampler (JAGS [33]) implemented in R [34]. We based our analyses on Bayesian posterior distributions of the factor of interest (i.e. the grouping variable for geolocator birds versus controls) so that probabilistic statements regarding the difference between geolocator birds and controls could be made. For each analysis we report the median value of the posterior distribution for the grouping variable, the 95% credible intervals (CI; lower/upper bound) and the probability that the direction of the effect is the same as that reported for the median estimate (i.e. positive or negative). Thus, for a positive group effect estimate where 98% of the posterior distribution values were above zero, we report P(geo>ctr)=98%. All reported results were sampled directly from the posterior distribution output from JAGS; each MCMC chain consisted of 20000 values derived from 250000 iterations with a thinning interval of 10 and a burn-in of 50000. In all analyses 3 chains were run with different initial values and convergence checked using the Gelman and Rubin diagnostic ([35]; using the ‘gelman.diag’ function in R) and visual inspection of the chains. Non-informative priors were used in all the linear models (betas~dnorm(0, 0.000001), tau~dgamma(0.0001, 0.0001)).

**Table 4:** Comparison of survival (return rate) and breeding parameters between geolocator (geo) and control (ctr) birds after one year.

	control		geolocator		GLM	P
	mean±SE	n	mean±SE	n	median (CI)	%
return rate (%)	44.5	164	29.7	37	-0.36 (-1.23/0.44)	81.2 (geo<ctr)
arrival date (days)	-1.47±1.14	34	2.51±3.09	10	3.47 (-1.76/8.71)	90.6 (geo>ctr)
egg laying date (days)	-2.52±0.79	54	2.37±1.30	9	6.34 (2.18/10.56)	99.8 (geo>ctr)
between-year change in egg laying date (days)	-1.51±1.47	19	8.54±3.90	7	5.97 (1.05/10.92)	99.1 (geo>ctr)
nest success (%)	82.8	58	33.3	9	-2.83 (-5.12/-0.87)	99.8 (geo<ctr)

Differences of geolocator vs. control birds predicted by Bayesian GLMs are presented as median and 95% credible interval (CI; lower/upper bound) of the posterior distribution for the grouping variable, with P showing the probability that the direction of the effect is the same as that reported for the median estimate. Presented mean±SE refer to raw data. Arrival and egg laying date were standardised for annual variation. Number of fledglings were considered only for successful nests. The between-year change in egg laying dates refers to the within-individual change in standardised egg laying dates year 2 – year 1.

## Results

### Physical effects

Mantle feathers that had been growing underneath the geolocator during moult showed signs of abrasion (broken tips) for most birds (Figure S2), with one bird also showing some rubbing-induced skin irritation under the geolocator. On five individuals there was evidence of skin irritation and crusting (but no open wounds) on the inside of the legs at the point of harness contact. Despite these physical changes, no returning geolocator birds were observed behaving differently from other birds, nor were the birds with the physical changes the ones most delayed in breeding time (see below). When accounting for tarsus length (size) and trapping date there was a trend for geolocator birds to have a slightly higher body mass at recapture than controls (GLM: geo(yes) median=1.18 g, 95% CI=-0.63/2.95, P(geo>ctr)=91.2%; raw data: geo: 23.9±0.4g, n=10, ctr: 23.7±0.6g, n=7). Sample size for control birds was low because we do not routinely re-trap previously ringed individuals.

### Survival

**Figure 1:**
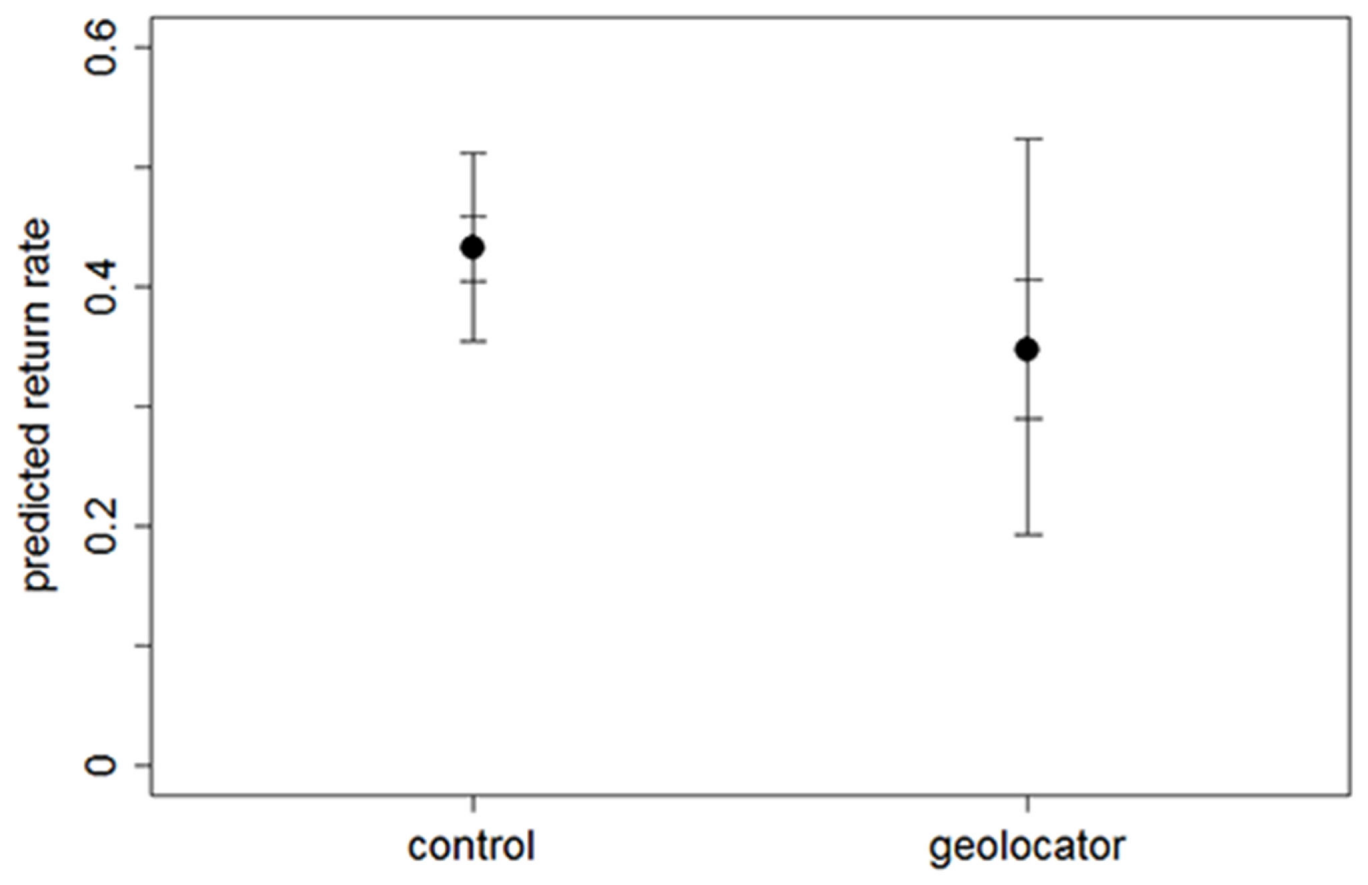
Return rates of control vs. geolocator birds. Predicted apparent survival (return rate) probability (median with 50% and 95% CI) of control (n=164) vs. geolocator (n=37) birds as predicted from Bayesian GLM with covariates as specified in the Methods.

Apparent survival (return rate) of geolocator birds tended to be lower than that of controls; with a 81% probability that geolocator birds had a lower return rate compared to controls when other factors known to influence between-year survival (see Methods) were controlled for (Table 4, Figure 1).

### Timing

**Figure 2:**
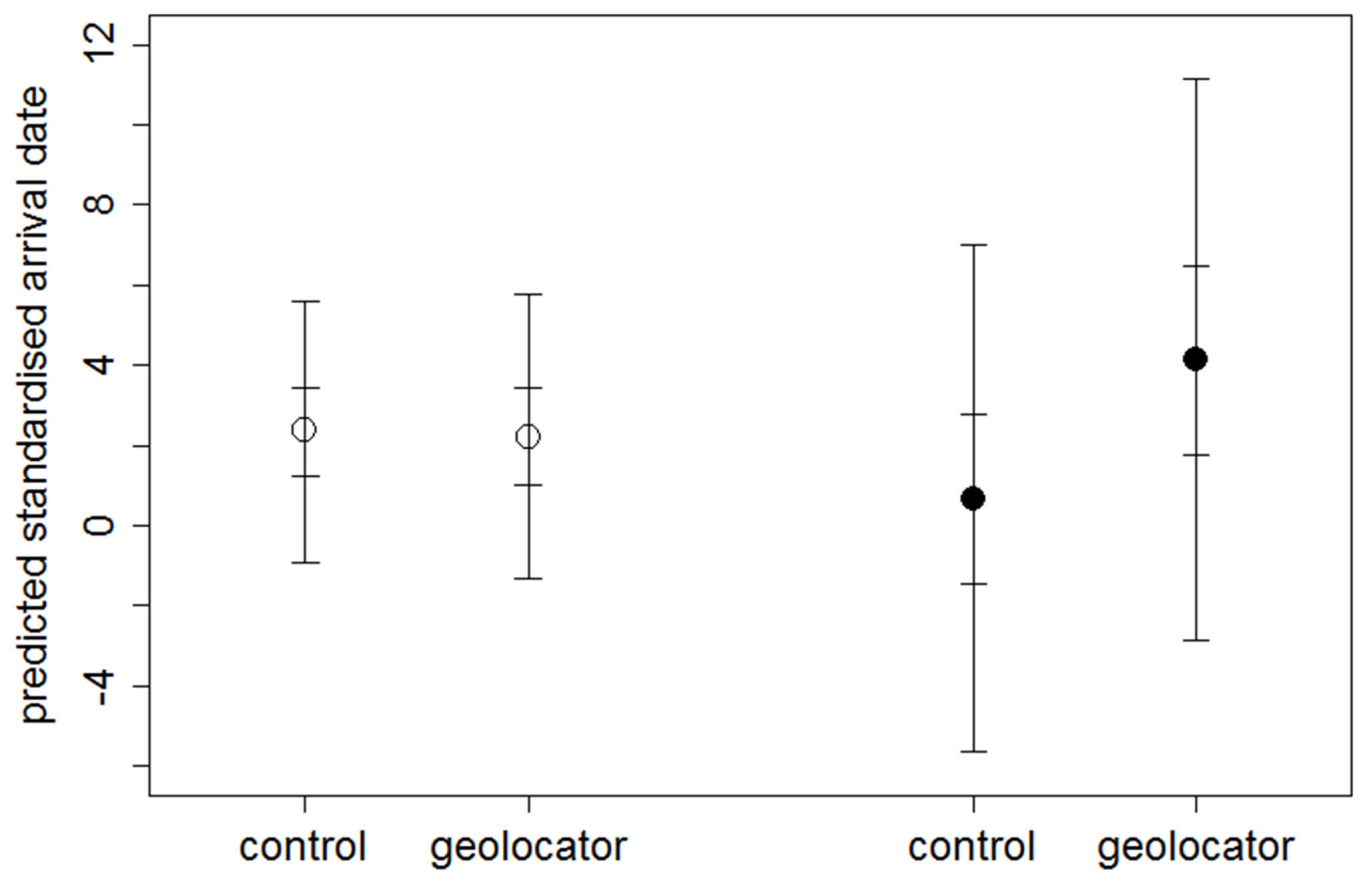
Arrival dates of control vs. geolocator birds. Model predictions (median with 50% and 95% CI) for standardised arrival dates of control vs. geolocator birds in the deployment (open symbols; n_ctr_=78, n_geo_=34) and post-deployment year (filled symbols; n_ctr_=34, n_geo_=10) from Bayesian GLM with covariates as specified in the Methods.

**Figure 3:**
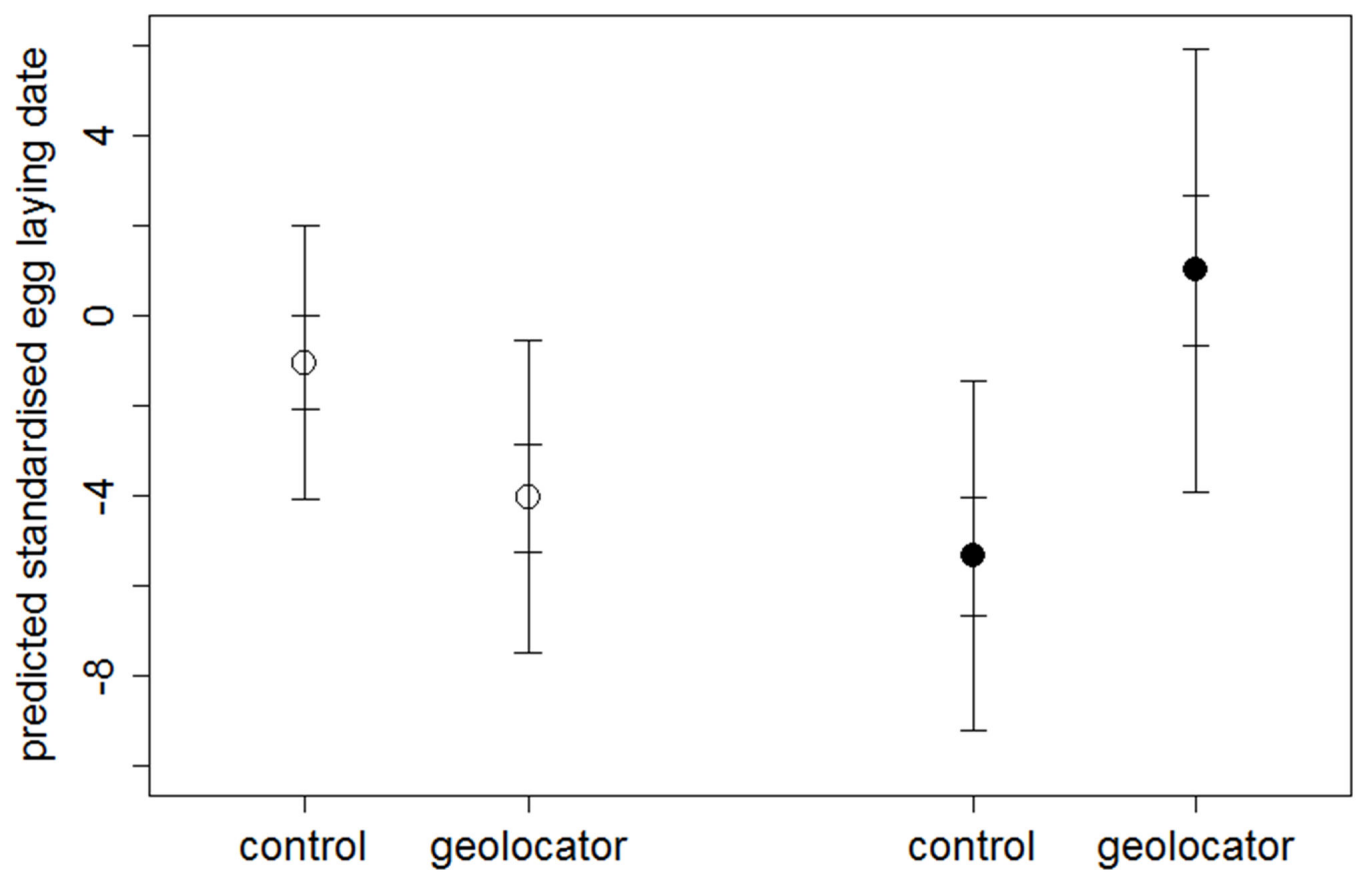
Egg laying dates of control vs. geolocator birds. Model predictions (median with 50% and 95% CI) for standardised egg laying dates of control vs. geolocator birds in the deployment (open symbols; n_ctr_=143, n_geo_=36) and post-deployment year (filled symbols; n_ctr_=54, n_geo_=9) from Bayesian GLM with covariates as specified in the Methods.

There was some support for geolocator birds arriving later than controls when accounting for an individual’s sex, age and previous year’s arrival date (Table 4, Figure 2), whereas there had been no difference in the deployment year (see above, Figure 2). Geolocator birds also initiated clutches later than control birds (Table 4, Figure 3), with the direction of this effect opposite to that seen in the deployment year (see above, Figure 3). Results were similar when restricting analyses to the smaller subset of individuals for which both arrival and breeding dates in both years were available (GLM: geo(yes) median=6.06 days, 95% CI=1.45/10.72, P(geo>ctr)=99.5%, n_geo_=8, n_ctr_=30). This difference was due to differences in within-individual between-year changes in timing of egg laying: compared to their own egg laying dates in the deployment year geolocator birds laid eggs later (difference in standardised egg laying date year 2 – year 1, Table 4). 

### Reproductive performance

Geolocator birds had lower nest success than controls (analysis without any covariates, geo: 25.0%, n=12, ctr: 78.1%, n=73; GLM: geo(yes) median=-2.43, 95% CI=-4.13/-1.01, P(geo<ctr)=99.9%). This difference remained after accounting for effects of field layer height and breeding time (Table 4). Nest failures among geolocator birds were mostly due to predation, in one case the nest was abandoned.

## Discussion

There was some support for geolocators negatively influencing survival and delaying arrival, in that there was an 81% probability that wheatears with geolocators had lower apparent survival than control birds (i.e. birds trapped but no geolocator mounted) and a 91% probability that wheatears with geolocators arrived later to the breeding grounds. The influence of geolocators on subsequent breeding parameters was much stronger, with a >99% probability that geolocator birds initiated clutches later and had lower breeding success than controls. Although our sample of geolocator birds is relatively small, each measure indicated that geolocator birds performed less well than birds without geolocators. Using the known breeding history of individuals these results are strengthened by observing the same negative effects for within-individual changes in performance before and after the deployment of the geolocator. Thus, our study demonstrates potential negative consequences of carrying a geolocator during a full year, in a bird that exhibits annual long-distance migration, for migratory performance and subsequent reproductive performance. 

Previous studies on effects on tracking devices [17,19,20] suggest that devices can induce behavioural changes, and energetic costs affecting various aspects of behaviour and ecology. Geolocator studies often lack data to rigorously test such effects. Studies that are not carried out within a population study usually lack data on control individuals. Reported effects vary: with some studies not finding effects on reproduction or survival [36-38], and others finding effects on breeding parameters and reproductive performance [39], or reporting low return rates (e.g. [8,38,40]). A recent review found no evidence of a general negative effect of geolocators on survival, although such an effect was evident in some individual studies [41]. Some studies have not investigated whether effects of geolocators occur (e.g. [6,42]). 

The geolocators that we used were just at the commonly used upper permissible load limits at the time of attachment (Table 1), and thus the relatively heavy load may partly explain the results. However, passerine geolocator loads are typically between 4-5% (e.g. [7-9,12,42]). Furthermore, even relatively small devices could affect performance, e.g., relatively light (≤1% of the body mass) devices can cause chronic stress when applied over long periods (i.e. several months or usually up to one year) [43] or affect hormonal stress response [44]. Geolocators have now become just light enough (applying the 5% rule) to use on many more species of passerines than has been possible so far. Thus, for many small passerine species (<20 g) targeted by researchers in the near future, geolocator loads will be likely close to the 5% load limit. Therefore, investigations of possible short- and long-term impacts (i.e. within a few weeks during the deployment season, and resulting from effects accumulated over the duration of several months or one year, respectively) of geolocators on breeding and migratory performance are clearly warranted. 

A major effect seen in this study was that geolocator birds initiated clutches later than controls. This was because geolocator birds delayed their clutch initiation following migration, i.e. after carrying the geolocator for almost one year, whereas control birds initiated clutches at a similar time in both years. A similar delay in nest initiation was also reported by Barron et al. [17] when reviewing effects of all types of tracking devices. Few geolocator studies report effects on breeding time, and those that do, report no effects (brown skua *Catharacta lonnberg* [45]; lesser kestrel *Falco naumanni* [36]; northern wheatear [46]). Similarly, a study on great reed warblers *Acrocephalus arundinaceus* [47] found no effects of geolocators on arrival dates. However, in those studies the geolocator was <1% of the body mass or sample size was small.

The difference between geolocator and control birds in clutch initiation date (about 6 days) was greater than the difference in arrival date (about 3 days). Thus, the later breeding of our wheatears with geolocators seems partly explained by a slightly later arrival, together with a similar delay between arrival and clutch initiation. These effects could result directly from increased energetic costs (flight costs) through increased drag and/or weight [19,20] with these costs either increasing migration duration and hence delaying arrival, or being carried over and resulting in a longer pre-breeding interval. Increased energetic costs may lead to more frequent and/or longer stopovers (either direct or indirect, e.g. poorer condition and therefore low quality stop-over sites, effects), accumulating over time and resulting in overall longer migration duration [14,48]. Since the migration behaviour of control birds is unknown we can only speculate about potential causes for the observed difference in timing. Longer migration duration seems supported by preliminary data from our geolocator birds indicating that later arrival was related to longer total time spend on migration after the departure from the wintering area (D Arlt & T Pärt, unpublished data), assuming that geolocator birds have a similar total migration distance as control birds. Alternatively, later arrival may result from greater total migration distance because of different wintering sites or migration routes. Preliminary data, however, indicate that geolocator birds overwinter in a narrow latitudinal range of the Sahel belt where wheatears find appropriate habitat conditions, and can take short routes crossing the Mediterranean Sea between Algeria and Southern France/Northern Italy (D Arlt & T Pärt, unpublished data), thus we think it is unlikely that in our study geolocator birds have significantly greater total migration distances than control birds. 

Increased flight costs may also affect energy reserves after migration and/or increased stress levels (e.g. [43]) with potential consequences for the pre-breeding interval [49]. Delayed breeding could also result indirectly via lower territory quality of late arriving birds. However, most geolocator birds bred at similar (with respect to field layer height, i.e. an important estimator of territory quality, see Methods and [26]) or sometimes the same sites as in the deployment year. In fact, in year two 77.8% (7 of 9) geolocator birds bred at territories with short field layers as compared to 68.5% (37 of 54) among control birds (same sample of birds as in analysis of egg laying dates).

Geolocator studies often seem to assume that flight costs are small and that there are no effects on migration, with the assumption of small flight costs being based on investigations of effects of radio transmitters [50-52]. However, some studies have shown transmitters to slow flight and increase work load [53]. Furthermore, compared to geolocators that are carried during a full year, radio-transmitters are usually carried during only a short time period, and therefore, such studies are not considering potential long-term effects through accumulated costs during e.g. long-distance migration. Our results are in accordance with Barron et al. [17] who included in their meta-analysis not only radio transmitters but also satellite transmitters and other types of data logger and concluded that the most substantial effects of tracking devices were markedly increased energy expenditure.

Geolocator birds had also lower nest success in the post-deployment year than control birds, and this effect was independent of differences in breeding date or potential differences in territory quality (territory field layer height [26]). Lower nest success was not a direct effect of carrying the geolocator during incubation or nestling provisioning because most (except three) birds were re-trapped and geolocators were removed before incubation. Almost all nest failures were caused by nest predation after hatching (one abandoned) and because most birds were re-trapped before incubation low nest success is unlikely to be an effect of the recapture procedure. Furthermore, trapping and ringing birds is not associated with nest predation ([25], T Pärt, unpublished data).

## Conclusion

Our results imply that effects of geolocators on individual performance should not be neglected. Such effects can arise as direct instrumentation effects (e.g. affecting survival, arrival date, or length of pre-breeding interval) or as indirect effects (e.g. late arrival may influence territory selection with effects on breeding parameters). Our study performed on individuals with a known breeding history shows that effects may be subtle but still can influence migratory performance and subsequent reproductive performance. Geolocator studies aim to study migration patterns and performance, but have so far not discussed how migration, as documented by geolocators, may deviate from migration of unmanipulated birds. If geolocators increase flight costs and impact migratory performance, as our results suggest, this has implications for geolocator-based studies of migratory strategies investigating migration speed, flight distances during migration legs, stopover and departure decisions (e.g. at geographical barriers) in response to fine-scale environmental cues varying on a daily basis (e.g. weather) during migration. Clearly, future geolocator studies need to investigate potential effects of carrying geolocators on migration. As geolocators get lighter and smaller any effects on performance are also likely to get smaller. However, for many small passerine species the geolocator loads will still be relatively high and it will be of paramount importance to identify and investigate effects of geolocators on migratory performance. By investigating individuals with a known breeding history one strategy may be to identify individuals that are most affected and consider such individual differences when investigating migratory strategy and performance. One measurable indicator of such effects could be arrival and breeding phenology in comparison to control individuals and in particular within-individual changes in arrival time and the timing of breeding. However, to increase knowledge about geolocator effects on migration strategies and performance those effects should also be investigated more directly, for example by comparing the migration of individuals carrying geolocators with different weights, e.g. in species with body mass >50 g permitting a variation in relative geolocator weight between 1% (or less) and 5%.

## Supporting Information

Figure S1 **Wheatear tagged with a geolocator.**
(TIF)

Figure S2 **Wheatear after geolocator removal.**
(TIF)
