# Supplementary figures and images for: Effect of Geolocators on Migration and Subsequent Breeding Performance of a Long-Distance Passerine Migrant
Source: PLoS One. 2013 Dec 4;8(12):e82316. doi: 10.1371/journal.pone.0082316 (PMC3852741; doi:10.1371/journal.pone.0082316)

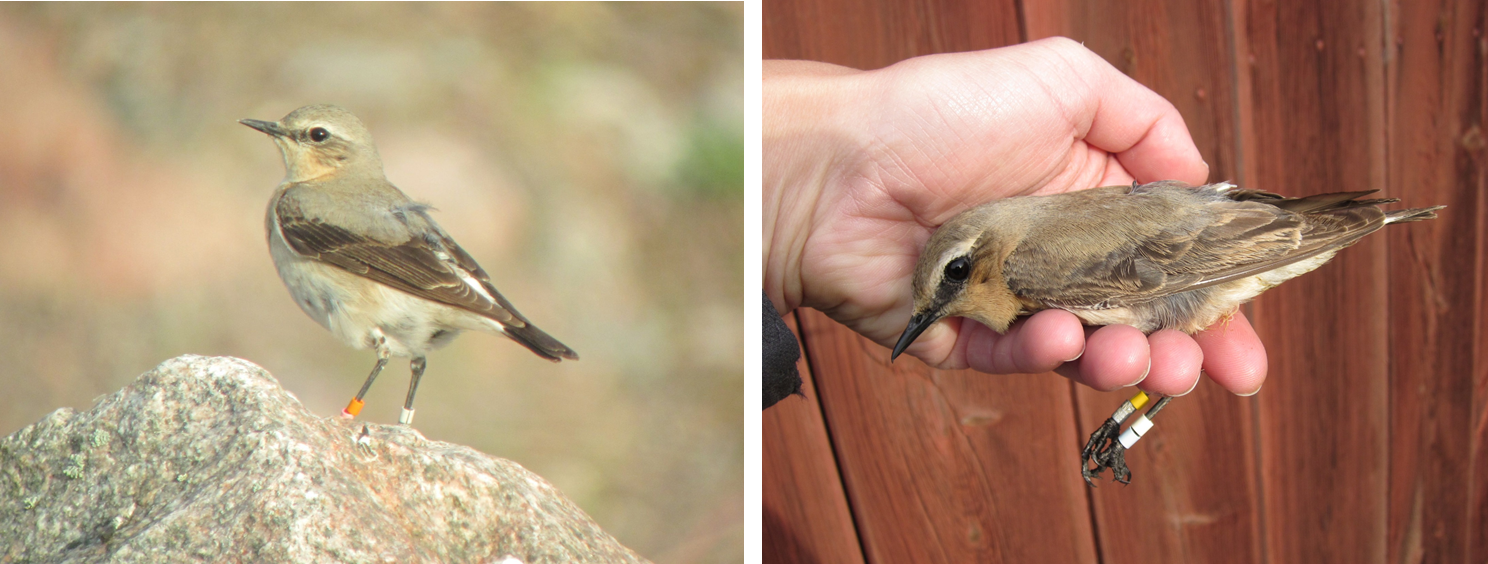

Supplement: Figure S1 — Wheatear tagged with a geolocator. (TIF) [file pone.0082316.s001.tif]

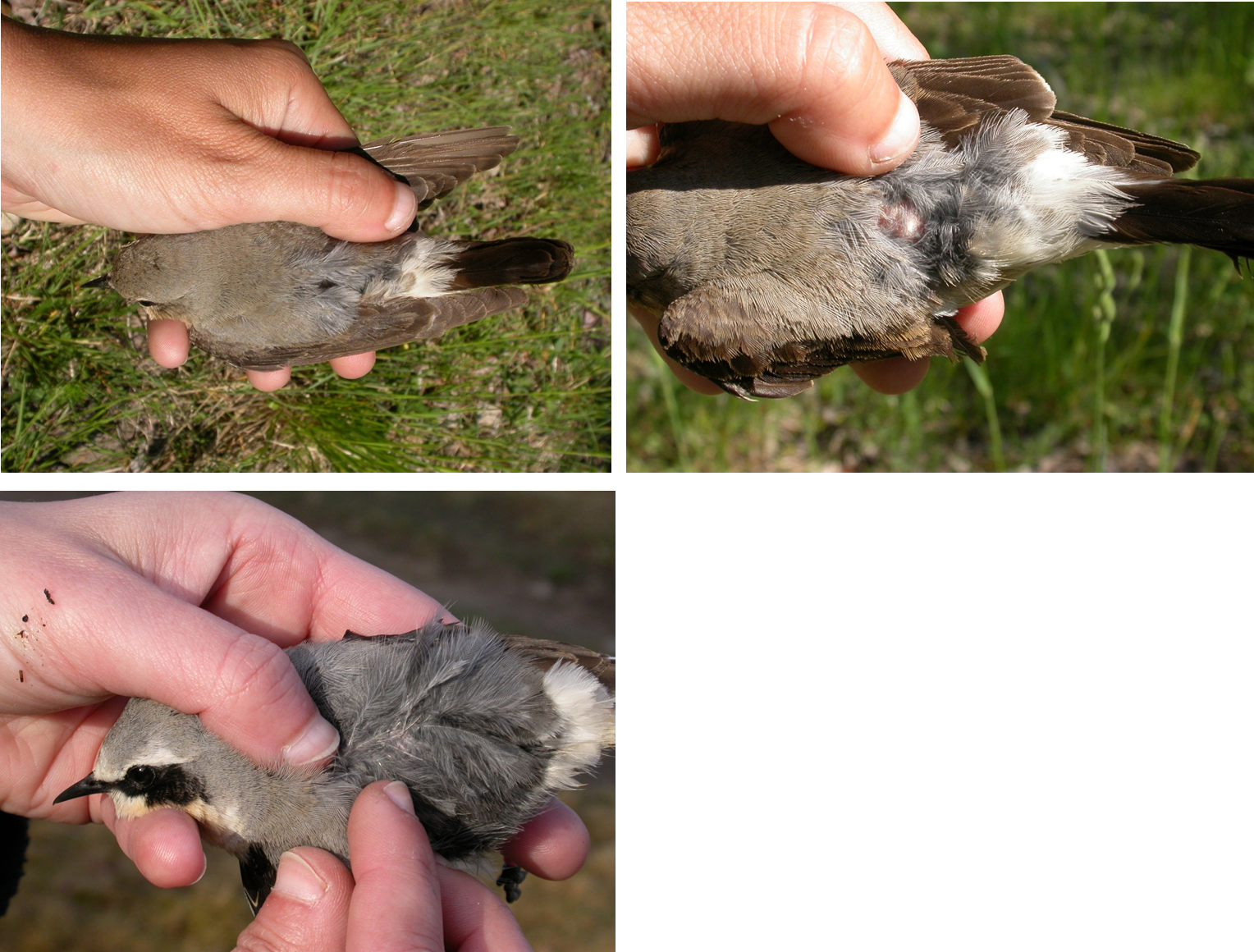

Supplement: Figure S2 — Wheatear after geolocator removal. (TIF) [file pone.0082316.s002.tif]
